# A case of percutaneous transhepatic portal vein stenting without using iodinated contrast media for post pancreatectomy portal vein obstruction

**DOI:** 10.1259/bjrcr.20220116

**Published:** 2023-10-27

**Authors:** Hinano Anaba, Sota Oguro, Hideki Ota, Satoru Yanagaki, Tomomi Sato, Kei Nakagawa, Michiaki Unno, Kei Takase

**Affiliations:** 1 Department of Diagnostic Radiology, Tohoku University Graduate School of Medicine, Sendai, Japan; 2 Department of Diagnostic Radiology, Tohoku University Hospital, Sendai, Japan; 3 Department of Surgery, Tohoku University Graduate School of Medicine, Sendai, Japan

## Abstract

Postoperative portal vein obstruction could occur as a complication of portal vein reconstruction during hepatic lobectomy or pancreaticoduodenectomy. We report a case of patient with postoperative portal vein obstruction treated with percutaneous transhepatic portal vein stenting without using iodinated contrast media owing to a history of severe allergic reactions. Under ultrasound guidance, carbon dioxide angiography, and appropriate device selection, successful stenting was achieved without serious adverse events. After the operation, portal vein blood flow and clinical symptoms improved, enabling adjuvant chemotherapy. To the best of our knowledge, this is the first case report wherein percutaneous transhepatic portal vein stenting was successfully performed in a patient with an iodine allergy.

## Introduction

Postoperative portal vein (PV) occlusion could arise from a surgical complication of PV reconstruction during hepatic lobectomy or pancreaticoduodenectomy.^1^ In recent years, percutaneous PV stenting has been considered as a treatment option. Iodinated contrast media is generally used during the procedure; however, its use is contraindicated in patients with a history of an allergic reaction, and alternative contrast media should be considered.

Herein, we report a case wherein carbon dioxide was used as a contrast media to treat a patient with long segmental obstruction of the portal venous system following total pancreatectomy who had a history of allergy to iodinated contrast media.

## Case report

**Figure 1.**
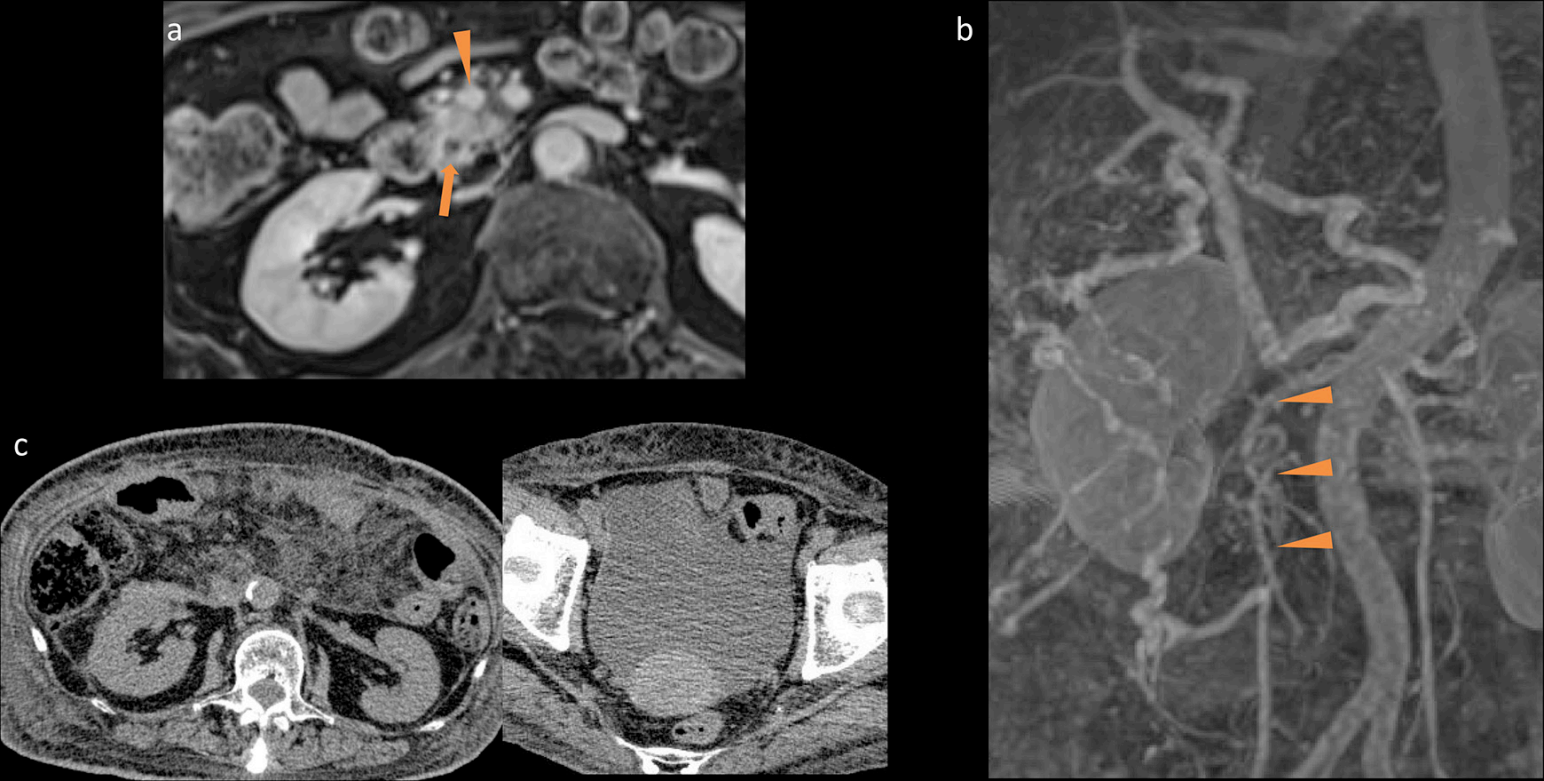
Contrast-enhanced magnetic resonance imaging (MRI). (**a**) Preoperative contrast-enhanced MRI revealed that the superior mesenteric vein (SMV) (arrowhead) was near the pancreatic cancer (arrow). (**b**) Postoperative contrast-enhanced MR angiography. A coronal maximum intensity projection image revealed a 6.5 cm portal vein and SMV occlusion (arrowhead). (**c**) Postoperative computed tomography showed massive ascites and oedema of the lower limbs.

A 74-year-old female underwent total pancreatectomy with partial resection of the superior mesenteric vein (SMV) for advanced pancreatic head carcinoma (Figure 1a). A curative resection was performed and adjuvant chemotherapy was initiated. However, chemotherapy was withdrawn due to ascites, diarrhoea, and oedema in the lower limbs. No obvious hepatic encephalopathy was observed. No varices were found. Cytological examination of the ascites did not provide any evidence of peritoneal dissemination. Gadolinium-based contrast-enhanced magnetic resonance imaging (MRI) demonstrated a 6.5-cm-long occlusion of the PV and SMV, with oedematous small intestine and ascites (Figure 1b). Portal hypertension secondary to PV occlusion was suspected. Angioplasty using a stent for the occluded PV and SMV was planned. However, the patient had a history of respiratory discomfort and disturbance of consciousness following iodinated contrast media injection, which was diagnosed as an allergic reaction. Therefore, carbon dioxide (CO_2_) was considered as an alternative to iodinated contrast media. Initially, the trans-ileocolic venous approach was attempted but was abandoned due to intra-abdominal adhesions. Thus, a percutaneous transhepatic portal venous approach was planned.

## Procedure

**Figure 2.**
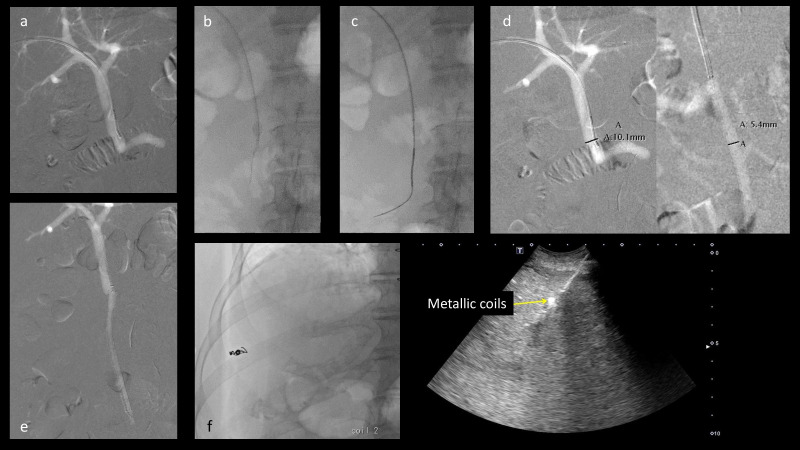
Percutaneous transhepatic portal vein stenting with carbon dioxide (CO_2_) angiography. (**a**) After a 4-Fr multipurpose pre-shaped catheter was advanced to the main portal vein (PV), angiography revealed that the superior mesenteric vein (SMV) and a portion of the PV were totally occluded. The left gastric vein was visualised. (**b**) The guidewire and the 4-Fr catheter were advanced approximately 1 cm into the occlusion. (**c**) After the micro-guidewire was passed through the occlusion, the catheter was advanced into the ileocolic vein. The 4-Fr catheter was advanced over the micro-catheter to reach the ileocolic vein. The 0.035-inch stiff straight wire was then inserted. (**d**) On CO_2_ angiography, the diameter of the main PV and ileocolic vein was 10.1 and 5.4 mm, respectively. A 6 mm × 10 cm self-expandable metallic stent was deployed from the ileocolic vein to the PV trunk. (**e**) Stent patency was confirmed with CO_2_ angiography. (**f**) The puncture route was embolised using two metallic coils under ultrasound guidance.

The intrahepatic PV (P5) was punctured under ultrasound guidance, and a 4-Fr sheath introducer (11 cm in length) was inserted. After a multipurpose pre-shaped catheter (4 Fr, 65 cm VTA, Medikit, Japan) was advanced to the main PV, digital subtraction angiography using CO_2_ gas was performed (Figure 2a). First, the advancement of a micro-catheter into the obstructed segment failed; thus, the segment was assumed to be hard. Therefore, the 11-cm 4-Fr sheath was replaced with a 45-cm-long 5-Fr guiding sheath (Destination, Terumo, Japan), for stronger reinforcement. A 0.035-inch guidewire could be advanced into the proximal part of the obstructed segment for 1.5 cm; the tip of the 4-Fr catheter was also advanced into this proximal part for 1 cm (Figure 2b). Second, the micro-catheter (2.6/2.8 Fr. Corsair, Asahi Intec, Japan) and a 0.014-inch micro-guidewire (Gladius, Asahi Intec, Japan) were used to attempt crossing over the occlusion. Finally, the micro-guidewire was passed through the occlusion, and the tip of the catheter was advanced into the ileocolic vein (Figure 2c). The 4-Fr catheter was advanced over the micro-catheter to reach the ileocolic vein, and a 0.035-inch stiff straight guidewire (Amplatz, Cook Medical, USA) was inserted. The 5-Fr sheath was replaced with a 6-Fr sheath (Destination, Terumo, Japan) compatible with stent deployment, which was advanced to the ileocolic vein. On CO_2_ angiography, the diameter of the main PV and ileocolic vein was 10.1 and 5.4 mm, respectively (Figure 2d). On preprocedural contrast-enhanced MRI, the length of the occlusion was 6.5 cm. Therefore, a self-expandable metallic stent (SMART control, Cordis, USA) with a size of 6 mm × 10 cm was deployed to cover the occlusion site, such that the proximal end of the stent protruded slightly into the PV trunk. We did not measure the pressure gradient across stenosis. Post-stenting balloon dilatation was performed using a 6 mm × 4 cm balloon catheter (Mustang, Boston Scientific, USA). After the patency of the stent was confirmed with CO_2_ angiography (Figure 2e), the puncture route was embolised using two 6/3-mm-sized metallic coils (Tornado, Cook Medical, USA) under ultrasonographic guidance (Figure 2f).

The ascites, diarrhoea, and oedema of the lower limbs improved, permitting resumption of adjuvant chemotherapy. No obvious changes in the liver enzyme levels were observed, while a mild increase in the albumin level was observed. Anticoagulation with aspirin and clopidogrel was administered for 6 months following the intervention. Follow-up ultrasonographic examination and MRI revealed in-stent patency to date.

## Discussion

In this case report, the long section (6.5 cm) PV occlusion was stented without using iodinated contrast media in a percutaneous transhepatic approach because the patient had a history of a severe allergy to iodine. CO_2_ was selected because it has been shown to be safe and effective in angiography. It is widely used in patients with renal impairment or iodine allergy. However, it results in poorer quality images compared with images obtained using iodinated contrast media.^2,3^ Although its use has been reported in percutaneous transhepatic portography, splenoportography,^2^ and renal artery stenting,^3^ this is the first case report of PV stenting using CO_2_ as contrast media.

Recanalisation of a long section of portal vein obstruction is often discouraged because of the associated technical difficulties; however, PV stenting was planned because of the physician and patient’s strong preference. Initially, an open surgical insertion (via the ileal vein) was planned because it was assumed that approaching the occluded PV from upstream would be easier than the percutaneous transhepatic approach. An open surgical approach is advantageous in that contrast media can be injected from upstream to check the condition of the stenosis or obstruction. However, it was impossible to place the sheath on the ileocolic vein due to intra-abdominal adhesions in our case. Therefore, the percutaneous transhepatic PV route was considered, which is reportedly more technically difficult but less invasive.^1,4–6^

The procedure was performed with ultrasound guidance and CO_2_ contrast. To overcome technical difficulties, device selection is crucial. First, a 4-Fr multipurpose pre-shaped catheter and 0.035-inch guidewire were used. However, it was still difficult to cross the occlusion due to chronic total occlusion. A stiffer guiding sheath was then used, which enabled crossing the occlusion; the device used in this procedure was a penetrating micro-system for the peripheral arteries.^7^ The ascites around the liver increased the risk of persistent bleeding following sheath removal. Hence, metallic coils were placed at the puncture site of the liver at the end of the procedure under ultrasound guidance.

Postoperative PV occlusion may be caused by surgical intervention (*e.g.,* PV reconstruction), recurrent malignancy, or radiation therapy. Olcott et al first reported stenting for PV stenosis in the anastomotic site of liver transplantation in 1990.^1,8,9^ Previous studies have suggested that percutaneous transhepatic PV stenting should be considered safe with few major complications.^1,4,5^ The technical success rates for benign PV stenosis were reportedly 76–100%,^10,11^ with symptom improvement observed in 75–100%, 80–100%, and 78–100% of cases of hepatic decompensation, variceal bleeding, and ascites, respectively.^8^ The average duration of patency is reportedly 30–67 months.^9^ On the other hand, in occlusions associated with malignant tumours, the success rate of the procedure is reported to be 80–100%,^6^ and symptom improvement is 87–100%, 80–100%, and 88–100% for hepatic decompensation, variceal bleeding, and ascites, respectively.^8^ However, the average patency period is only 3.7–6.1 months, resulting in relatively early obstruction.^9^ Malignant PV obstruction is associated with advanced cancer and has a poor prognosis. Nevertheless, symptomatic improvement may contribute to a better prognosis if anticancer therapy can be resumed.

Considering the size of the stent, the vascular calibres were significantly different between the PV trunk and ileocolic vein: 10.1 and 5.4 mm, respectively. The appropriate size of the stent should be the same as the diameter of the non-stenotic vein or 1–2 mm larger. If the reference vessel diameters are different proximal and distal to the stenosis, it is safe to conform to the diameter of the smaller vessel. In our case, owing to the long occlusion length, a 6 mm × 10 cm stent was selected as the best available. A covered stent may be used in cases of stenosis attributed to malignancy; however, the risk of occlusion or stenosis of the portal tributaries exists.^6^ In our case, a bare stent would be better because the stenosis was suspected to be attributed to an adhesion. Taourel et al reported that portal velocity or flow on Doppler measurement had limited utility in predicting either hepatic venous pressure gradients or severity of liver failure in an individual patient because of the scattering of the data.^12^ When portal hypertension is due to cirrhosis, even an implanted PV stent may not provide good blood flow and may soon become occluded. It is not possible to predict whether the PV pressure is elevated, and it is better to measure the pressure at the SMV upstream. Even if the pressure is elevated, stenting should be performed. Due to possible stent occlusion, bare-metal stents should be used to cover necessary and sufficient area.

Postoperative medication is crucial, considering that many patients with cancer are in a state of hypercoagulability. The efficacy of anticoagulant drugs following PV stenting is controversial. Although various case reports^4,13^ of anticoagulants administered alone or in addition to antiplatelet drugs exist, there was one report wherein anticoagulation was not used.^5^ In our case, stent flow was prompt upon ultrasound examination; thus, an anticoagulant drug was not administered. Patency was maintained for at least 1 year.

## Conclusion

We report a case of percutaneous transhepatic PV stenting for postoperative PV obstruction without using iodinated contrast media in a patient with iodine allergy. With ultrasound guidance, CO_2_ angiography, and appropriate device selection, successful stenting was achieved without serious complications, allowing the patient to resume adjuvant chemotherapy.

## Learning Point

- CO2 contrast media is useful for portal stenting in patients with iodine allergy.
